# The use of digital tools in rare neurological diseases towards a new care model: a narrative review

**DOI:** 10.1007/s10072-024-07631-4

**Published:** 2024-06-10

**Authors:** Francesca Torri, Gabriele Vadi, Adriana Meli, Sara Loprieno, Erika Schirinzi, Piervito Lopriore, Giulia Ricci, Gabriele Siciliano, Michelangelo Mancuso

**Affiliations:** 1 Department of Clinical and Experimental Medicine, Neurology Unit, University of Pisa, Pisa, Italy; 2 Department of Translational Research and New Technologies in Medicine and Surgery, University of Pisa, Pisa, Italy

**Keywords:** Rare neurological diseases, Digital tools, Wearable devices, Reachable workspace, Gait analysis, Digital outcome measures

## Abstract

Rare neurological diseases as a whole share peculiar features as motor and/or cognitive impairment, an elevated disability burden, a frequently chronic course and, in present times, scarcity of therapeutic options. The rarity of those conditions hampers both the identification of significant prognostic outcome measures, and the development of novel therapeutic approaches and clinical trials. Collection of objective clinical data through digital devices can support diagnosis, care, and therapeutic research. We provide an overview on recent developments in the field of digital tools applied to rare neurological diseases, both in the care setting and as providers of outcome measures in clinical trials in a representative subgroup of conditions, including ataxias, hereditary spastic paraplegias, motoneuron diseases and myopathies.

## Introduction

**Fig. 1:**
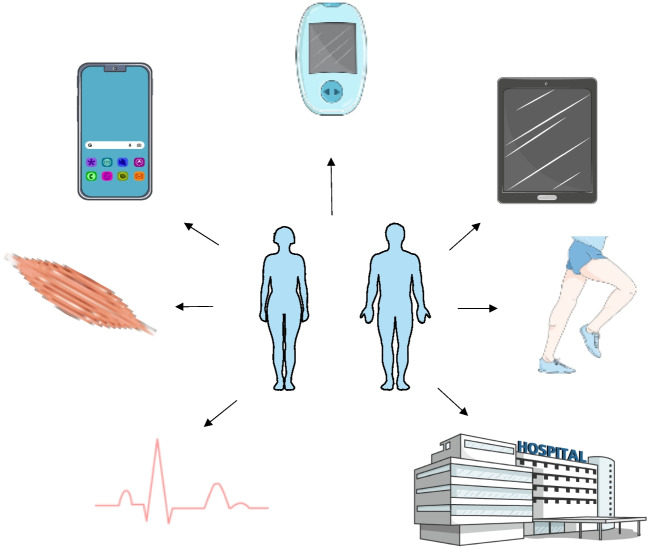
Graphical representation of the components and subjects of application of digital tools for tele-monitoring, digital data collection and clinical trials design. Created with SMART – Servier Medical Art (https://smart.servier.com/)

Although numerous and heterogenous in clinical presentation, rare neurological diseases (RND) represent a specific entity, sharing peculiar features as motor and/or cognitive impairment, an elevated disability burden, a frequently chronic course and, in present times, scarcity of therapeutic options [1]. The rarity of those conditions and their variability, often without a straightforward genotype–phenotype correlation, hampers both the identification of significant prognostic outcome measures, and the development of novel therapeutic approaches and clinical trials. Given also the global economic burden of RNDs, mainly imputable to the slow progression and the presence of multiple comorbidities (respiratory, cardiological, rehabilitation and orthopedics, infections, nutrition, and many others) [1], the optimization of care and the identification of sensitive outcome measures is paramount. In this setting, the development and application of digital tools, may they be registers, wearable or tele-health devices, represents a transformative paradigm potentially providing accuracy, durability, and homogeneity of data collection. For example, in a clinical field where many patients display moderate to severe degree of motor disability and logistics for specialist visits are not always comfortable for them and for caregivers, digital tools can provide a continuous measurement of selected biological parameters, as movement, sleep, respiratory and cardiological data (Fig. 1). Collecting this information in a prolonged manner also better depicts disease progression, as subtle changes may go unnoticed in periodic visits. For the same reason, realizing clinical trials, which are mainly held in few reference centers and often include patients from different geographical sites of the country, could face less accessibility problems and guarantee a wider recruitment. Finally, in recent times digital outcome measures are contributing to efficacy assessments in clinical trials and have been validated by regulatory agencies [2]. Lessons have been learned also from the COVID-19 pandemics, that halted the possibility of many patients to access regular evaluations due to emergency travel restrictions, quarantine, and fear of contagion [3]. These events prompted the development and implementation in clinical practice of telehealth systems [3]. This review aims at providing an overview on the panorama of the use of digital tools (biosensors, wearable devices, video-recordings, and others)in supporting clinical management and pharmaceutic trials in RNDs, as myopathies, motoneuron diseases (MND), ataxias and hereditary spastic paraplegias (HSP).

## Methods

This review was conducted searching for the latest publications regarding the application of digital tools in rare neurological diseases, both in clinical practice and trials. Therefore, ee searched on the PubMed and ClinicaTrials.gov databases using the following keywords: “digital tools”, “wearable devices”, “biosensors”, “actimyo”, “ALS”, “SMA”, muscular dystrophy”, “myopathy”, “pompe disease”, “ataxia”, “HSP”, “6mwt”, “SARA scale” and papers from the last five years were prioritized. We did not consider papers written in other languages than English. Retrospective studies, prospective observational studies and clinical trials were included in the selection. We included reports from ClinicalTrials.gov mentioning digital tools in clinical trials which have yet to begin or have not published final results, given the novelty of the subject specifically in RNDs, and in order to provide an updated description of the practical state of the art in this context.

## Digital tools applied to care and clinical trials in RNDs

### Ataxias and HSPs

Ataxias are a heterogeneous group of movement disorders due to damage of the cerebellum and related brain structures, which are characterized by the inability to harmoniously coordinate the sequential activation of the muscle groups predominately involved in the coordination of balance, gait, speech, and limb and eye movements, resulting in loss of balance, the inability to maintain posture and perform movements or actions in a coordinated manner [4]. Hereditary spastic paraplegias are a heterogeneous group of neurodegenerative disorders, secondary to the pyramidal tracts involvement, which are characterized by progressive lower-limb spasticity resulting in a spastic and/or ataxic gait, leading to disability and increased risk of falls [5]. Biomechanically, ataxic gait is characterized by decreased speed and step length along with increased step width and variability in the timing and direction of steps [6], while paraparetic gait is characterized by reduced ranges of motion in lower body joints, which limit foot clearance and ankle range of motion [7]. Moreover, in ataxia, postural dysfunction consists in increased body sway and base width during standing as well as during walking [8]. Currently, ataxia and HSP severity and progression are commonly assessed using clinical rating scales [9–13]. The most used clinical score for ataxic disorders is the Scale of the Assessment and Rating of Ataxia (SARA), while the Spastic Paraplegia Rating Scale (SPRS) is the widely used and well-accepted scale for HSP severity assessment [11, 12]. However, clinical scales depend on the subjective assessment by clinicians [12, 13]. Therefore, quantifiable markers of motor function, which allow a more objective detection of subtle changes and feasible quantitative measurements, are increasingly being explored as useful tools to be applied for clinical and experimental applications in neurology and neurorehabilitation. In this regard, in the recent years, novel technologies, including computer interfaces, videogames or “serious games” and wearable sensors have emerged [14].

#### Gait analysis

Abnormalities in spatiotemporal gait parameters, such as reduced speed and step length, increased base width, and increased variability of step features, are consistently identified as the most significant feature of gait patterns in cerebellar ataxia [15]. Gait impairment in hereditary spastic paraparesis patients includes several abnormalities, such as reduced step length, increased step width, reduced range of motion at the knee joint, impaired knee torque and stiffness, and decreased activity of the rectus femoris muscle [16–18]. Therefore, gait analysis is a valuable source of clinical biomarkers both in ataxia and HSP, providing accurate quantitative assessments of patients throughout their entire disease course [19–21]. Gait parameters obtained from optical motion capture systems, including range of motion of ankle, knee, and hip joints and foot clearance, may be important and disease-specific digital biomarkers of HSP [7, 16, 22]. They seem to correlate with the severity of the disease, reflecting the wide clinical heterogeneity of gait disorders between subgroups of patients with different severity [7, 22]. Similar data come from studies in which the assessment of paraparetic gait was performed by wearable inertial measurement units (IMU) [23, 24], and it has been shown that gait parameters reflected relevant information, such as the SPRS [23], or the fear of falling and quality of life [24]. Wearable inertial sensors have shown to be sensitive and specific to simultaneously assess gait and balance also in patients with cerebellar ataxia [25, 26]. Moreover, a recent study has demonstrated that combining data from wearable sensors with machine learning allows to accurate predict of dysfunction progression in individual patients with FA, and molecular cause of the disease (repressed Frataxin) from movement data alone [27]. The advantage of wearable sensors systems is that they allow gait analysis even in the non-clinical environment, allowing to assess patients’ gait continuously in their everyday life [28, 29]. Promising results in assessing movement in ataxia appear to come from the Microsoft Kinect v2 sensor, a low-cost RGB-D camera [30]. Kinect-based gait analysis system has proven to be effective in deriving spatial–temporal gait parameters reliably in ataxic patients, such as speed, stride length and the step length, that could be promising technology-based biomarkers to assess and follow-up patients into a clinical setting [30]. Moreover, also the RGB-depth camera-based motion analyses of mediolateral truncal sway during walking and arrhythmicity of stepping in place could be useful digital motor biomarkers for the assessment of cerebellar ataxia and could be utilized in future clinical trials [14]. Indeed, the lack of local trunk stability is a major feature in cerebellar ataxia [6], therefore, quantify patterns of trunk acceleration during gait in ataxic patients could be a useful tool for clinicians. The mediolateral amplitude of truncal sway during walking, assessed by triaxial accelerometers attached at the upper back of the patients, has been shown to correlate well with severity of ataxia and fall risk, and has been proposed as a potential biomarker of gait impairment and as well reliable predictor of risk of fall in patients with cerebellar ataxia [31–34].

#### Speech analysis

In recent years, accurate assessment methods to assess dysarthria begin to be explored, since the dysarthria is a common and debilitating symptom of ataxia [35]. Computer-assisted analysis of speech allows an objective assessment, including features that are not accessible to the hearing of the human examiner. Grobe-Einsler et al. have developed an automated assessment of ataxic speech system, the SARA speech, which has demonstrated to be able to predict the severity of speech disturbance [36].

#### Remote monitoring

The advent of “Internet of Medical Things” (IoMT) and of the Information Communication Technology (ICT) have further revolutionized the traditional healthcare systems. These systems allow the remote control of the novel technological devices and the real-time communication of data with clinicians, by connecting to healthcare information technology systems using safe networking technologies [37, 38]. Zilani et al. have proposed a method for monitoring patients with ataxia using ultra-wide band (UWB) technology to track movements during walking in indoor environment [37]. Summa et al. have developed a low-cost and innovative system, integrating Kinect, LMC and IoMT technologies, which allows the remote, standardized, and objective quantification of ataxia, in young patients with cerebellar ataxia, even in non-hospital settings [38].

#### Ocular movements analysis

Summa et al. suggest saccades as potential biomarkers in cerebellar ataxia [39]. The pattern of saccadic impairment seems to differ between different type of ataxia, reflecting distinct pathophysiological substrates. The assessment of saccadic impairment by a non-invasive video-oculography device, consisting in a head- mounted system of miniaturized cameras, has shown that vertical saccades are prominent involved in cerebellar ataxia, while the horizontal ones in FRDA [39]. Chang et al. have developed a new inexpensive and widely accessible system for quantifying abnormalities in smooth pursuit in individuals with ataxia using a mobile device camera to record eye movements while viewing stimuli on a tablet screen. This system combined with signal processing and machine learning techniques, can accurately and rapidly detect abnormalities in smooth pursuit and grade the severity of oculomotor dysfunction in cerebellar ataxias [40]. Therefore, video-oculography techniques could be valid tools to test saccades non-invasively and could be used to track severity of oculomotor impairment in clinical and trials setting.

#### Digital tools in clinical trials for *ataxia* and HSP

With disease-modifying drugs forthcoming for ataxias, there is a critical need for objective, reliable, sensitive, and specific outcome measures for upcoming trials to capture early disease progression and response to therapies [41]. Efforts are, therefore, ongoing to use new technological innovations to capture objective longitudinal data on patient symptoms. These include the use of wearable sensors or smartphones to capture movements, allowing for remote long‐term tracking of patients’ movements to describe the full range and variability of symptoms more accurately on a daily basis [42]. Digital gait and balance measures are both valid markers of disease progression and treatment response, therefore should be considered promising candidate outcomes measure for future clinical trials, as established in some observational trials [42–44]. Variability measure of gait and balance have been shown to correlate well with disease severity both in ataxia [31–33] and HSP [7, 22–24]. Wearable IMU sensor technology has been identified as the most appropriate technology at this time to conduct such multicenter studies of digital gait and balance measures in ataxia [45], having shown to be able to sensitive and specific to simultaneously assess gait and balance [25, 26]. Wearable IMU sensor technology has also demonstrated to be valid for the assessment of paraparetic gait [23, 24] and was used for gait analysis in several studies [29, 46]. Other technologies used for the assessment of gait analysis in HSP include infrared multi-camera motion analysis system [22, 47], pressure sensors [48], and 3D motion analysis [17, 49]. Recently, Ollenschläger et al. have proposed a system for measure HSP gait cycles using an automated and continuous assessment of phenotypical disease features in terms of reduced foot elevation by standardized gait tests using wearable sensors and machine learning classifiers [29]. Regarding ataxia, as previously mentioned, potential biomarkers for future trial include mediolateral trunk-sway which well correlates with gait impairment and risk of fall [14, 31, 33, 34], and spatio-temporal gait parameters (speed, stride length and the step length) which best reflecting gait features in ataxic patients [30].

Digital tools applied to ataxias and HSP are summarized in Table 1.

**Table 1:** Summary of digital tools applied to care and clinical trials in ataxias/HSP

Digital tool	Disease	Analyzed parameter
Optical motion capture systems	Ataxia/HSP	Gait abnormalities
Wearable IMU	Ataxia/HSP	Gait abnormalities
RGB-depth camera-based motion analyses (Microsoft Kinect 2.0)	Ataxia	Gait abnormalities, truncal sway
Triaxial accelerometers	Ataxia	Truncal sway
Computer-assisted voice assessment and dysarthria classification	Ataxia	Dysarthria
UWB technology	Ataxia	Remote monitoring of gait
Microsoft Kinect 2.0 and Leap Motion Controller (LMC)—SaraHome	Ataxia	Performance of items included in the SARA scale
Non-invasive video-oculography	Ataxia	Saccades impairment

### Motorneuron diseases (ALS and SMA)

In the emerging era of telemedicine and machine learning, clinical monitoring takes on new and dynamic perspectives. In this scenario, new technologies could play a crucial role in clinical monitoring, and in the case of motor neuron disorders, this concept gains even more significance with the advent of novel therapies. Currently, we are witnessing a transformative therapeutic era in spinal muscular atrophy (SMA) marked by the widespread use of newly approved disease-modifying therapies. The introduction of such drugs, coupled with the possibility of early administration, has already brought about a profound change in the clinical landscape and the overall history of SMA [50]. While remarkable results have been observed in children, monitoring outcomes in adults proves more challenging and only limited data from clinical trials in adult SMA is available [51]. In this context, the use of new technologies in clinical monitoring becomes crucial for capturing subtle differences often overlooked in assessments performed in adults but mostly designed for children.

#### Upper limb evaluation

A study demonstrated the potential of the Microsoft Kinect sensor to address the critical need for objective and sensitive outcome measures in SMA. The sensor, through a specifically designed game, captured upper limb movement allowing detailed analysis of joint motion limitations in a cohort of SMA III patients. This technology could serve as a complementary output measure for SMA, offering reproducible, objective, and detailed information on body point motion [52]. Another study utilized the ActiMyo device to measure physical activity in non-ambulant SMA patients. The device effectively recorded upper limb movements in daily life, revealing decreased wrist vertical acceleration in sitting SMA Type 2 patients compared to non-sitters [53]. This information has the potential to assess fatigue and loss of endurance during daily activities, crucial for measuring quality of life impairment. As a matter of fact, fatigue is particularly difficult to detect, and as it’s often reported as improved by adult patients undergoing therapies, efforts are ongoing to effectively measure such characteristic. For example, a recent study evaluated the effect of wearable devices for the assessment of surface EMG and joints positions and angles for upper and lower limbs while performing a new functional motor scale specifically designed for evaluating the endurance dimension and were overall well tolerated [54].

#### Speech assessment

In the case of amyotrophic lateral sclerosis (ALS), efforts are particularly directed towards anticipating disease evolution and progression, as early detection of disease progression can be crucial for timely interventions. One study employed a mobile application to assess speech features in ALS patients, revealing that biomarkers obtained from the app could detect disease progression earlier than Revised Amyotrophic Lateral Sclerosis Functional Rating Scale (ALSFRS-R) bulbar scores [55]. Another study proved to detect early speech changes and track speech progression in ALS via automated algorithmic assessment of speech collected remotely [56]. Clinical instruments for assessing bulbar function often lack sensitivity to early changes, and the incorporation of new technologies could provide valuable insights [57].

#### Remote monitoring

Physical activity through biotelemetry was assessed in another study as clinical measure of ALS progression; this method proved as a direct, objective, and real-life assessment of physical function, highlighting its potential as a clinical monitoring tool [58]. Overall, the development of digital biomarkers enables home tele-management, allowing medical staff to evaluate patients from a distance and provide counselling and therapeutic intervention at the appropriate time. Digital health technology has the potential to enhance care accessibility and personalization, while remote biosensors can optimize the gathering of crucial clinical parameters, regardless of patients' ability to physically visit a clinic [59].

#### Digital tools in clinical trials for motorneuron diseases

New technologies can already provide substantial benefits in clinical trials even in the field of motor neuron disorders. Undoubtedly, the COVID-19 pandemic accelerated the adoption of remote monitoring along with the use of digital tools, wearable devices, and telehealth solutions to facilitate patients in maintaining contact with physicians from their homes, especially with a focus on detecting disease progression [60]. Remote and decentralized trials could increase accessibility, reduce the burden on participants, and allow for a more diverse and inclusive participant pool [61], while using devices for remote data collection also minimizes the necessity for participants to attend frequent trial appointments[62, 63], helping in overcoming barriers related to patient acceptance and adherence[64]. The use of accelerometers, for example, proved to be an objective means to measure disease progression, providing valuable real-world insights into a patient's physical functioning[65]. This data has the potential to personalize the delivery of care[65] and could represent potential real-world information to be integrated with traditional clinical trial, offering insights into long-term effectiveness and safety.

The potential application of digital technologies in this context may extend beyond specialized biosensors to include smartphones themselves. A smartphone sensor-based assessment for patients with SMA, demonstrated strong reliability, validity, and feasibility in measuring motor and muscle function[66]. Self-reporting data through smartphone app represent a powerful tool as well. Self-administered ALSFRS-R scores obtained through smartphones exhibit a strong correlation with clinic-based ALSFRS-R scores, demonstrating minimal variability and suggesting their potential utility in clinical trials [61]. In a randomized trial that compared remote nutritional counselling with or without mobile health technology for ALS patients, the Nu Planit application was deemed acceptable and useful as a mobile app for monitoring nutritional status [67].

Machine learning can significantly contribute to improving and adding an extra edge to new clinical trials. A recent study used an analytical method that automatically identifies and characterizes sub-movements based on accelerometer data passively collected, producing a machine-learned severity score for each limb: this approach produced scores progressing faster compared to the established ALSFRS-R, leading to smaller clinical trial sample size estimates [68]. The authors concluded that this method could offer a scalable measure for potential use in amyotrophic lateral sclerosis trials. Johnson et al. in a recent investigation concluded that the utilization of both active (surveys) and passive (sensors) digital data collection through mobile apps and wearable devices shows potential for the development of innovative outcome measures in ALS trials [69].

A recent survey showed that the majority of patients with MND expressed a favorable outlook on the utilization of digital technology in both healthcare and clinical trial contexts[70]; however, a subset of patients raised concerns about home-monitoring, underscoring the need to address these doubts and to provide the adoption of proper measures. Among this concerns privacy plays an important role and every effort should be made to ensure that all data are collected securely and in accordance with specific regulations. Finally, and not least, among new technologies “omics sciences” are rapidly transforming clinical studies of MNDs, offering a more comprehensive understanding of molecular pathways, and enabling the categorization of patients into diagnostic, prognostic, and therapeutic subgroups, marking a crucial juncture in the evolution of personalized medicines for MNDs[71].

Digital tools applied to motorneuron diseases are summarized in Table 2.

**Table 2:** Summary of digital tools applied to care and clinical trials in motorneuron diseases

Digital tool	Disease	Analyzed parameter
Microsoft Kinect sensor	SMA	Upper limb movement
ActiMyo	SMA	Upper limb movement
Surface EMG and accelerometers	SMA	Motor endurance of upper and lower limbs
Mobile speech analysis	ALS	Dysarthria
Wrist-worn ActiGraph and ankle-worn StepWatch 4	ALS	Remote movement monitoring
Smartphone sensor-based assessment suite	SMA	Motor function
Mobile app	ALS	Nutritional counseling

### Myopathies

Myopathies are rare diseases primarily affecting muscles, mainly genetic but also secondary to autoimmune diseases, metabolic imbalances, and other conditions. With regards to inherited forms, thousands of genetic variants are known, and clinical manifestations can have a wide range of age at onset, progression rate, phenotype, and eventual multisystem involvement [72]. Moreover, after decades of conservative treatment based on rehabilitation and symptomatic therapies, disease-modifying drugs are being developed and the number of clinical trials has skyrocketed. In this perspective, the use of digital tools in myopathies could provide a great support in diagnosis, clinical reasoning, and development of clinical trials.

#### Gait and walking activity analysis

Several devices have been applied to different muscle disease: Hamed et al. [73] explored the use of a wearable tracker to analyze median step count and peak 1-min activity in 29 adult, ambulatory Late-onset Pompe disease (LOPD) patients. LOPD is a rare genetic disorder caused by mutations in the *GAA* gene, coding for the acid alpha-glucosidase enzyme resulting in slowly progressing limb-girdle weakness and respiratory involvement [74]. The authors reported high engagement in data sharing from patients. The digitally acquired parameters were confronted with fatigue and pain dimensions through patient-reported questionnaires and showed a direct relationship. In Duchenne muscular dystrophy (DMD), the most common muscular dystrophy in childhood [75], several tools have been applied to assess motor function and other parameters. Xiong et al. [76] recently investigated the gait pattern of 20 patients with DMD compared to control children by 3D Gait Analysis and describe several parameters as stride length, percentage of stance and swing phase, step length, and percentage of double support phase. The authors report significant differences between the two groups in gait velocity, stride length, and step length, concluding that the symmetry of synergistic movement of lower limbs is impaired in DMD and can discriminate between DMD and control children.

#### Respiratory and autonomic dysfunction analysis

For instance, a study is ongoing on DMD boys to assess glucose blood levels and heartrate variability as measures of autonomic dysfunction [77], information of great interest in a cohort of patients chronically treated with steroids as disease modifying therapy [78]; data about motor activity and sleep duration will be acquired through accelerometers. Other studies focused on digital monitoring of respiratory function [79] through inertial measurement units (IMU-based devices) for continuous monitoring of breathing frequency in patients with DMD and limb-girdle muscular dystrophy (LGMD), which is an indicator of respiratory difficulty when increased.

#### Multiparametric evaluation

Another muscle disease in which digital assessment tools have been applied is Facioscapulo-humeral Muscular Dystrophy (FSHD). A study investigated the use of smartphones and wearable devices to collect data as number of steps, sleep, and whether patients would be keen on using the app. The authors report a positive attitude from patients towards the app, which allowed for the collection of daily activity information and social behavior of subjects involved [80]. Mellion et al. [81] correlated whole body muscle MRI data to functional parameters, including reachable workspace (RWS), to be used as outcome measures in therapeutic clinical trials. RWS is a digital measure obtained by recording of movement range of upper limbs in space, thus providing a quantified 3D representation [82]. This tool has been applied to FSHD, DMD [83], ALS [84] and other conditions and has been included as outcome measure in the Fulcrum clinical trials, phases II and III, to assess the efficacy of losmapimod in FSHD [85]. In this clinical trial, patients were recruited based on the genetic diagnosis of FSHD and a certain range of disability, without considering the well-known phenotypic variability in this disease, so that also the use of a digital outcome measure as RWS can become difficult to interpret. Gerhalter et al. [86] developed suMus, a smartwatch app equipped with motor exercises videos. Performances are recorder by the inertial sensors of the smartwatch and after each session information feedback from the patient is asked. Many patients involved in the study were affected by muscular dystrophies along with congenital and myofibrillar myopathies and other neuromuscular diseases. Also, Myotonic Dystrophy type I, characterized by a predominant distal impairment of upper and lower limb muscles, is used as a model to apply wearable devices. A study from Duong et al. will assess the standard-of-care motor outcome measures with the aid of wearable devices and video-recordings of myotonia of the hands (Video Hand Opening Time—VHOT) [87]. Ricotti et al. [88] developed a sensor equipped bodysuit for DMD patients to study movement behavior and applied machine learning approaches to combine the identified behavioral hallmarks of DMD subjects and developed a behavioral biomarker, termed the KineDMD ethomic biomarker, able to predict progression and sense treatment response. Wearable devices equipped with surface electromyography (EMG) and accelerometers, named AUTOMA, have been developed for motor assessment of upper limbs also by Milazzo et al. [89].

#### Remote rehabilitation

Wearable devices and tele-monitoring could be a helpful tool also in rehabilitation, as proved by a trial on Duchenne patients that underwent telerehabilitation sessions for five weeks through virtual reality glasses to train for the treatment goals, with increase in 6MWT and timed up and go test, while the Motor Function Measure in all of the 3 dimensions showed no significant differences as well as the North Start Ambulatory Assessment (NSAA) scores. This study suggested the non-inferiority of telerehabilitation treatment compared to conventional sessions and could be helpful in facilitating access to therapies [90].

#### Digital tools in clinical trials for myopathies

Wearable devices and digital outcome measures are increasingly applied to clinical trials to demonstrate the efficacy of new treatments also in muscular dystrophies. A gene therapy clinical trial for DMD [91] will be assessing change from baseline captured through interactive video evaluation (ACTIVE)—Seated Workspace Volume, in Stride and Stair-climbing Velocity, in number of stairs, distance walked per hour and angular wrist velocity, assessed by a wearable device. A similar approach will be used in a gene therapy trial for Limb Girdle Muscular Dystrophy, Type 2E/​R4 (Beta-Sarcoglycan Deficiency) [92]. Stride velocity 95th centile (SV95C) deserves a special mentioning, being the first digital clinical outcome measure, wearable-derived, approved by the European Medicines Agency (EMA) for use as a secondary endpoint in trials for Duchenne muscular dystrophy in 2019 [2]. It provides real-world assessment of ambulation peak performance over 180 h of recording, and it has been demonstrated to correlate with conventional clinical evaluations and motor scales.

Digital tools applied to myopathies are summarized in Table 3.

**Table 3:** Summary of digital tools applied to care and clinical trials in myopathies

Digital tool	Disease	Analyzed parameter
Wearable tracker	LOPD	Steps number
Accelerometers	DMD	Glucose level, heart rate, quantity of movement and sleep
IMU based sensors	DMD, LGMD	Respiratory function
3D Gait Analysis	DMD	Gait
Mobile sensors and wearable devices	FSHD	Number of steps, sleep
RWS	FSHD	Upper limbs Motor function
SuMus Mobile app	Myopathies	Remote training
VHOT	Myotonic Dystrophy type 1	Hand myotonia
Virtual reality glasses	DMD	Telerehabilitation
Sensor-equipped bodysuit	DMD	Motor behavior
Wearable devices	DMD	SV95C, Number of stairs, distance walked per hour and angular wrist velocity

## Possible pitfalls in the use of digital tools in RNDs

Rare neurological diseases in general share critical aspects, including the relatively small number of affected subjects, their heterogeneity, the uncertainty of genotype–phenotype correlations and the difficulties encountered in general and specialist care. Digital tools could provide answers to these matters, if properly applied. The primary limitation of the available evidence nowadays is the small number of subjects generally included in the studies. In the analyzed studies the average number of involved affected subjects is of 20 or 30, which is a relatively scarce number considering the rarity of conditions but also hampers the possibility to apply subgroups analysis (for instance considering phenotypes, gender, age ranges, treatment status). Moreover, whenever a measuring tool capable of detecting very small, discrete changes is applied, the correct selection of patients to include in studies becomes paramount, in order to avoid misinterpretation of the obtained results, both in observational studies and clinical trials. Thus, to strengthen the use of digital tools and validate some of them as outcome measures, they should be at first applied on large, clinically, and genetically homogeneous cohorts. Further, once data is obtained, their meaning should be correctly evaluated: especially for tools providing very precise measurements, as the one assessing saccades, dysarthria, surface EMG and others, what should be considered as the yield of significance of results? In fact, if for already standardized tests like the 6MWT the use of a digital tool can simplify data acquisition and analysis, for other measurements also the normality range or the meaning of variations through time should be established. Again, this prompts the need to test the available systems on large cohorts of properly selected patients. Finally, the implementation of digital tools, often web based, in clinical practice and trials, must confront the privacy and data protection issue. The most recent European GDPR 2016/679 [93] regulation imposes certain standards that need to be implemented by all center that wish to use digital tools, and this may require adaptation procedures of the available hardware and software systems commonly used. Moreover, the development of artificial intelligence (AI) systems to data analysis acquired through digital tools adds further questions to consider, including the learning phases of algorithms which should be carried out on large amounts of properly collected data; even after that phase, nonetheless, the GDPR regulation itself denies the possibility to take decisions solely based on results from the application of AI [93].

## Conclusions

Rare neurological diseases are facing paramount changes in global care and therapeutical development. The improvement in genetic diagnosis leads to a rising number of cases and phenotypes, which in turn contribute to shed light to the physiopathology of diseases and pose challenges for natural history description and understanding, and for the identification of suitable outcome measures to apply to clinical trials. Moreover, although rare, these diseases affect a considerable number of patients, which often need to refer to few specialized centers far from home. Data collection through tele-monitoring, wearable devices and digital tools in general is contributing to the development of a new model of care and research, but must confront data protection and transmission issues, technical reliability of devices and accurate validation processes. Digital tools will represent in future years a significant element in assisting patients with RNDs and in the search for novel therapies.

## References

[1] Reinhard C, Bachoud-Lévi AC, Bäumer T et al (2021) The European Reference Network for Rare Neurological Diseases. Front Neurol 14(11):616569. 10.3389/fneur.2020.616569 10.3389/fneur.2020.616569 PMC7840612 33519696

[2] Servais L, Yen K, Guridi M et al (2022) Stride Velocity 95th Centile: Insights into Gaining Regulatory Qualification of the First Wearable-Derived Digital Endpoint for use in Duchenne Muscular Dystrophy Trials. J Neuromuscul Dis 9(2):335–346. 10.3233/JND-210743 34958044 10.3233/JND-210743 PMC9028650

[3] Mauri E, Abati E, Musumeci O et al (2020) Estimating the impact of COVID-19 pandemic on services provided by Italian Neuromuscular Centers: an Italian Association of Myology survey of the acute phase. Acta Myol 39(2):57–66. 10.36185/2532-1900-008 32904925 10.36185/2532-1900-008 PMC7460733

[4] Bodranghien F et al (2016) Consensus Paper: Revisiting the Symptoms and Signs of Cerebellar Syndrome. Cerebellum 15(3):369. 10.1007/S12311-015-0687-3 26105056 10.1007/s12311-015-0687-3 PMC5565264

[5] Shribman S, Reid E, Crosby AH, Houlden H, Warner TT (2019) Hereditary spastic paraplegia: from diagnosis to emerging therapeutic approaches. Lancet Neurol 18(12):1136–1146. 10.1016/S1474-4422(19)30235-2 31377012 10.1016/S1474-4422(19)30235-2

[6] Chini G et al (2017) Local stability of the Trunk in patients with degenerative cerebellar ataxia during walking. Cerebellum 16(1):26–33. 10.1007/S12311-016-0760-6 26811155 10.1007/s12311-016-0760-6

[7] Serrao M et al (2016) Gait patterns in patients with hereditary spastic paraparesis. PLoS One 11(10). 10.1371/JOURNAL.PONE.0164623 10.1371/journal.pone.0164623 PMC5061421 27732632

[8] Serrao M et al (2012) Gait pattern in inherited cerebellar ataxias. Cerebellum 11(1):194–211. 10.1007/S12311-011-0296-8 21717229 10.1007/s12311-011-0296-8

[9] Trouillas P et al (1997) International cooperative ataxia rating scale for pharmacological assessment of the cerebellar syndrome. J Neurol Sci 145(2):205–211. 10.1016/S0022-510X(96)00231-6 9094050 10.1016/s0022-510x(96)00231-6

[10] Schmahmann JD, Gardner R, MacMore J, Vangel MG (2009) Development of a brief ataxia rating scale (BARS) based on a modified form of the ICARS. Mov Disord 24(12):1820–1828. 10.1002/MDS.22681 19562773 10.1002/mds.22681 PMC3800087

[11] Schmitz-Hübsch T et al (2006) Scale for the assessment and rating of ataxia: development of a new clinical scale. Neurology 66(11):1717–1720. 10.1212/01.WNL.0000219042.60538.92 16769946 10.1212/01.wnl.0000219042.60538.92

[12] Schüle R et al (2006) The Spastic Paraplegia Rating Scale (SPRS): a reliable and valid measure of disease severity. Neurology 67(3):430–434. 10.1212/01.WNL.0000228242.53336.90 16894103 10.1212/01.wnl.0000228242.53336.90

[13] Weyer A et al (2007) Reliability and validity of the scale for the assessment and rating of ataxia: a study in 64 ataxia patients. Mov Disord 22(11):1633–1637. 10.1002/MDS.21544 17516493 10.1002/mds.21544

[14] Suzuki M et al (2023) Digital motor biomarkers of cerebellar ataxia using an RGB-Depth camera-based motion analysis system. Cerebellum. 10.1007/S12311-023-01604-7 37721679 10.1007/s12311-023-01604-7

[15] Buckley E, Mazzà C, McNeill A (2018) A systematic review of the gait characteristics associated with Cerebellar Ataxia. Gait Posture 60:154–163. 10.1016/J.GAITPOST.2017.11.024 29220753 10.1016/j.gaitpost.2017.11.024

[16] Klebe S et al (2004) Gait analysis of sporadic and hereditary spastic paraplegia. J Neurol 251(5):571–578. 10.1007/S00415-004-0366-7 15164190 10.1007/s00415-004-0366-7

[17] Piccinini L, Cimolin V, D’Angelo MG, Turconi AC, Crivellini M, Galli M (2011) 3D gait analysis in patients with hereditary spastic paraparesis and spastic diplegia: a kinematic, kinetic and EMG comparison. Eur J Paediatr Neurol 15(2):138–145. 10.1016/J.EJPN.2010.07.009 20829081 10.1016/j.ejpn.2010.07.009

[18] Marsden J, Ramdharry G, Stevenson V, Thompson A (2012) Muscle paresis and passive stiffness: Key determinants in limiting function in Hereditary and Sporadic Spastic Paraparesis. Gait Posture 35(2):266. 10.1016/J.GAITPOST.2011.09.018 22050971 10.1016/j.gaitpost.2011.09.018 PMC3657152

[19] Ilg W et al (2016) Individual changes in preclinical spinocerebellar ataxia identified via increased motor complexity. Mov Disord 31(12):1891–1900. 10.1002/MDS.26835 27782309 10.1002/mds.26835

[20] Rochester L, Galna B, Lord S, Mhiripiri D, Eglon G, Chinnery PF (2014) Gait impairment precedes clinical symptoms in spinocerebellar ataxia type 6. Mov Disord 29(2):252–255. 10.1002/MDS.25706 24301795 10.1002/mds.25706 PMC6551218

[21] Vasco G et al (2016) Functional and gait assessment in children and adolescents affected by Friedreich’s Ataxia: a one-year longitudinal study. PLoS One 11:(9). 10.1371/JOURNAL.PONE.0162463 10.1371/journal.pone.0162463 PMC5012652 27598307

[22] Laßmann C et al (2022) Specific gait changes in prodromal hereditary spastic paraplegia Type 4: preSPG4 Study. Mov Disord 37(12):2417–2426. 10.1002/MDS.29199 36054444 10.1002/mds.29199

[23] Regensburger M et al (2022) Inertial gait sensors to measure mobility and functioning in hereditary spastic paraplegia: a cross-sectional multicenter clinical study. Neurology 99(10):E1079–E1089. 10.1212/WNL.0000000000200819 35667840 10.1212/WNL.0000000000200819 PMC9519248

[24] H. Gaßner et al (2021) Functional gait measures correlate to fear of falling, and quality of life in patients with Hereditary Spastic Paraplegia: A cross-sectional study. Clin Neurol Neurosurg 209. 10.1016/J.CLINEURO.2021.106888 10.1016/j.clineuro.2021.106888 34455170

[25] Shah VV et al (2021) Gait variability in spinocerebellar ataxia assessed using wearable inertial sensors. Mov Disord 36(12):2922–2931. 10.1002/MDS.28740 34424581 10.1002/mds.28740

[26] Zhou H et al (2022) Assessment of gait and balance impairment in people with spinocerebellar ataxia using wearable sensors. Neurol Sci 43(4):2589–2599. 10.1007/S10072-021-05657-6 34664180 10.1007/s10072-021-05657-6

[27] Kadirvelu B et al (2023) A wearable motion capture suit and machine learning predict disease progression in Friedreich’s ataxia. Nat Med 29(1):86–94. 10.1038/s41591-022-02159-6 36658420 10.1038/s41591-022-02159-6 PMC9873563

[28] Ilg W et al (2020) Real-life gait assessment in degenerative cerebellar ataxia: Toward ecologically valid biomarkers. Neurology 95(9):E1199–E1210. 10.1212/WNL.0000000000010176 32611635 10.1212/WNL.0000000000010176

[29] Ollenschläger M et al (2023) Automated assessment of foot elevation in adults with hereditary spastic paraplegia using inertial measurements and machine learning. Orphanet J Rare Dis 18(1):249. 10.1186/S13023-023-02854-8 37644478 10.1186/s13023-023-02854-8 PMC10466820

[30] Summa S et al (2020) Validation of low-cost system for gait assessment in children with ataxia. Comput Methods Programs Biomed 196. 10.1016/J.CMPB.2020.105705 10.1016/j.cmpb.2020.105705 32846316

[31] Shirai S et al (2019) The responsiveness of triaxial accelerometer measurement of gait ataxia is higher than that of the scale for the assessment and rating of ataxia in the early stages of spinocerebellar degeneration. Cerebellum 18(4):721–730. 10.1007/S12311-019-01025-5 30993540 10.1007/s12311-019-01025-5

[32] Shirai S, Yabe I, Matsushima M, Ito YM, Yoneyama M, Sasaki H (2015) Quantitative evaluation of gait ataxia by accelerometers. J Neurol Sci 358(1–2):253–258. 10.1016/J.JNS.2015.09.004 26362336 10.1016/j.jns.2015.09.004

[33] Castiglia SF et al (2023) Identification of gait unbalance and fallers among subjects with cerebellar ataxia by a set of trunk acceleration-derived indices of gait. Cerebellum 22(1):46–58. 10.1007/S12311-021-01361-5 35079958 10.1007/s12311-021-01361-5

[34] Caliandro P et al (2019) Exploring risk of falls and dynamic unbalance in cerebellar ataxia by inertial sensor assessment. Sensors (Basel) 19(24). 10.3390/S19245571 10.3390/s19245571 PMC6960492 31861099

[35] Vogel AP et al (2020) Features of speech and swallowing dysfunction in pre-ataxic spinocerebellar ataxia type 2. Neurology 95(2):E194–E205. 10.1212/WNL.0000000000009776 32527970 10.1212/WNL.0000000000009776

[36] Grobe-Einsler M et al (2023) SARAspeech-Feasibility of automated assessment of ataxic speech disturbance. NPJ Digit Med 6(1). 10.1038/S41746-023-00787-X 10.1038/s41746-023-00787-x PMC10020430 36927996

[37] Zilani TA, Al-Turjman F, Khan MB, Zhao N, Yang X (2020) Monitoring Movements of Ataxia Patient by Using UWB Technology. Sensors (Basel) 20(3). 10.3390/S20030931 10.3390/s20030931 PMC7039007 32050576

[38] Summa S et al (2020) Development of SaraHome: A novel, well-accepted, technology-based assessment tool for patients with ataxia. Comput Methods Programs Biomed 188. 10.1016/J.CMPB.2019.105257 10.1016/j.cmpb.2019.105257 31846831

[39] Summa S et al (2020) A wearable video-oculography based evaluation of saccades and respective clinical correlates in patients with early onset ataxia. J Neurosci Methods 338. 10.1016/J.JNEUMETH.2020.108697 10.1016/j.jneumeth.2020.108697 32205159

[40] Chang Z et al (2020) Accurate detection of cerebellar smooth pursuit eye movement abnormalities via mobile phone video and machine learning. Sci Rep 10(1). 10.1038/S41598-020-75661-X 10.1038/s41598-020-75661-x PMC7596555 33122811

[41] Ilg W et al (2023) Quantitative Gait and Balance Outcomes for Ataxia Trials: Consensus Recommendations by the Ataxia Global Initiative Working Group on Digital-Motor Biomarkers. Cerebellum. 10.1007/S12311-023-01625-2 37955812 10.1007/s12311-023-01625-2 PMC11269489

[42] Brooker SM, Edamakanti CR, Akasha SM, Kuo SH, Opal P (2021) Spinocerebellar ataxia clinical trials: opportunities and challenges. Ann Clin Transl Neurol 8(7):1543–1556. 10.1002/ACN3.51370 34019331 10.1002/acn3.51370 PMC8283160

[43] Klockgether T et al (2022) Paving the Way Toward Meaningful Trials in Ataxias: An Ataxia Global Initiative Perspective. Mov Disord 37(6):1125–1130. 10.1002/MDS.29032 35475582 10.1002/mds.29032

[44] Ilg W et al (2022) Digital gait biomarkers allow to capture 1-year longitudinal change in spinocerebellar ataxia type 3. Mov Disord 37(11):2295–2301. 10.1002/MDS.29206 36043376 10.1002/mds.29206

[45] Walton MK et al (2020) Considerations for development of an evidence dossier to support the use of mobile sensor technology for clinical outcome assessments in clinical trials. Contemp Clin Trials 91. 10.1016/J.CCT.2020.105962 10.1016/j.cct.2020.105962 32087341

[46] Loris E et al (2023) Mobile digital gait analysis objectively measures progression in hereditary spastic paraplegia. Ann Clin Transl Neurol 10(3):447–452. 10.1002/ACN3.51725 36622133 10.1002/acn3.51725 PMC10014001

[47] Bonnefoy-Mazure A, Turcot K, Kaelin A, De Coulon G, Armand S (2013) Full body gait analysis may improve diagnostic discrimination between hereditary spastic paraplegia and spastic diplegia: a preliminary study. Res Dev Disabil 34(1):495–504. 10.1016/J.RIDD.2012.09.005 23085499 10.1016/j.ridd.2012.09.005

[48] Van Lith BJH, Den Boer J, van de Warrenburg BPC, Weerdesteyn V, Geurts AC (2019) Functional effects of botulinum toxin type A in the hip adductors and subsequent stretching in patients with hereditary spastic paraplegia. J Rehabil Med 51(6):434–441. 10.2340/16501977-2556 30968942 10.2340/16501977-2556

[49] van de Venis L, van de Warrenburg B, Weerdesteyn V, Geurts ACH, Nonnekes J (2023) Gait-adaptability training in people with hereditary spastic paraplegia: a randomized clinical trial. Neurorehabil Neural Repair 37(1):27–36. 10.1177/15459683221147839 36695288 10.1177/15459683221147839 PMC9896539

[50] Ludolph AC, Wurster CD (2019) Therapeutic advances in SMA. Curr Opin Neurol 32(5):777–781. 10.1097/WCO.0000000000000738 31425176 10.1097/WCO.0000000000000738

[51] Sansone VA et al (2020) Measuring Outcomes in Adults with Spinal Muscular Atrophy - Challenges and Future Directions - Meeting Report. J Neuromuscul Dis 7(4):523–534. 10.3233/JND-200534 32538864 10.3233/JND-200534

[52] Chen X et al (2017) Feasibility of using microsoft kinect to assess upper limb movement in type III spinal muscular atrophy patients. PLoS One 12(1). 10.1371/JOURNAL.PONE.0170472 10.1371/journal.pone.0170472 PMC5266257 28122039

[53] Chabanon A et al (2018) Prospective and longitudinal natural history study of patients with Type 2 and 3 spinal muscular atrophy: Baseline data NatHis-SMA study. PLoS One 13(7). 10.1371/JOURNAL.PONE.0201004 10.1371/journal.pone.0201004 PMC6062049 30048507

[54] Ricci G et al (2023) Proposal of a new clinical protocol for evaluating fatigability in adult SMA patients. Acta Myol 42(2–3):65–70. 10.36185/2532-1900-330 38090548 10.36185/2532-1900-330 PMC10712654

[55] Stegmann GM et al (2020) Repeatability of commonly used speech and language features for clinical applications. Digit Biomark 4(3):109–122. 10.1159/000511671 33442573 10.1159/000511671 PMC7772887

[56] Stegmann GM et al (2020) Early detection and tracking of bulbar changes in ALS via frequent and remote speech analysis. NPJ Digit Med 3(1). 10.1038/S41746-020-00335-X 10.1038/s41746-020-00335-x PMC7555482 33083567

[57] Ball LJ, Beukelman DR, Pattee GL. Timing of speech deterioration in people with amyotrophic lateral sclerosis. J Med Speech Lang Pathol. 10(4):231–235, 2002. [Online]. Available: https://experts.nebraska.edu/en/publications/timing-of-speech-deterioration-in-people-with-amyotrophic-lateral. Accessed 30 Dec 2023

[58] Kelly M et al (2020) The use of biotelemetry to explore disease progression markers in amyotrophic lateral sclerosis. Amyotroph Lateral Scler Frontotemporal Degener 21(7–8):563–573. 10.1080/21678421.2020.1773501 32573278 10.1080/21678421.2020.1773501

[59] Van Eijk RPA et al (2021) A road map for remote digital health technology for motor neuron disease. J Med Internet Res 23(9). 10.2196/28766 10.2196/28766 PMC8495582 34550089

[60] Bombaci A et al (2021) Telemedicine for management of patients with amyotrophic lateral sclerosis through COVID-19 tail. Neurol Sci 42(1):9–13. 10.1007/S10072-020-04783-X 33025327 10.1007/s10072-020-04783-x PMC7538170

[61] Berry JD et al (2019) Design and results of a smartphone-based digital phenotyping study to quantify ALS progression. Ann Clin Transl Neurol 6(5):873–881. 10.1002/ACN3.770 31139685 10.1002/acn3.770 PMC6529832

[62] Beswick E et al (2022) A systematic review of digital technology to evaluate motor function and disease progression in motor neuron disease. J Neurol 269(12):6254. 10.1007/S00415-022-11312-7 35945397 10.1007/s00415-022-11312-7 PMC9363141

[63] Rutkove SB et al (2020) Improved ALS clinical trials through frequent at-home self-assessment: a proof of concept study. Ann Clin Transl Neurol 7(7):1148–1157. 10.1002/ACN3.51096 32515889 10.1002/acn3.51096 PMC7359124

[64] Meyer T et al (2023) Remote digital assessment of amyotrophic lateral sclerosis functional rating scale - a multicenter observational study. Amyotroph Lateral Scler Frontotemporal Degener 24(3–4):175–184. 10.1080/21678421.2022.2104649 35912984 10.1080/21678421.2022.2104649

[65] van Eijk RPA, Bakers JNE, Bunte TM, de Fockert AJ, Eijkemans MJC, van den Berg LH (2019) Accelerometry for remote monitoring of physical activity in amyotrophic lateral sclerosis: a longitudinal cohort study. J Neurol 266(10):2387–2395. 10.1007/S00415-019-09427-5 31187191 10.1007/s00415-019-09427-5 PMC6765690

[66] Perumal TM et al (2023) Digital measures of respiratory and upper limb function in spinal muscular atrophy: design, feasibility, reliability, and preliminary validity of a smartphone sensor-based assessment suite. Neuromuscul Disord 33(11):845–855. 10.1016/j.nmd.2023.07.008 37722988 10.1016/j.nmd.2023.07.008

[67] Wills AM et al (2019) Nutritional counseling with or without mobile health technology: A randomized open-label standard-of-care-controlled trial in ALS. BMC Neurol 19(1):1–9. 10.1186/S12883-019-1330-6/FIGURES/3 31142272 10.1186/s12883-019-1330-6 PMC6540456

[68] Gupta AS, Patel S, Premasiri A, Vieira F (2023) At-home wearables and machine learning sensitively capture disease progression in amyotrophic lateral sclerosis. Nat Commun 14(1):1–12. 10.1038/s41467-023-40917-3 37604821 10.1038/s41467-023-40917-3 PMC10442344

[69] Johnson SA et al (2023) Wearable device and smartphone data quantify ALS progression and may provide novel outcome measures. NPJ Digit Med 6(1). 10.1038/S41746-023-00778-Y 10.1038/s41746-023-00778-y PMC9987377 36879025

[70] Helleman J et al (2022) Patient perspectives on digital healthcare technology in care and clinical trials for motor neuron disease: an international survey. J Neurol 269(11):6003–6013. 10.1007/S00415-022-11273-X/FIGURES/5 35849154 10.1007/s00415-022-11273-x PMC9294855

[71] Ruffo P, Cavallaro S, Conforti FL (2022) The Advent of Omics Sciences in Clinical Trials of Motor Neuron Diseases. J Personalized Med 12(5):758. 10.3390/JPM12050758 10.3390/jpm12050758 PMC9144989 35629180

[72] Mercuri E, Bönnemann CG, Muntoni F (2019) Muscular dystrophies. Lancet 394(10213):2025–2038. 10.1016/S0140-6736(19)32910-1 31789220 10.1016/S0140-6736(19)32910-1

[73] Hamed A, Curran C, Gwaltney C, DasMahapatra P (2019) Mobility assessment using wearable technology in patients with late-onset Pompe disease. NPJ Digit Med 22(2):70. 10.1038/s41746-019-0143-8 10.1038/s41746-019-0143-8 PMC6646308 31341956

[74] Taverna S, Cammarata G, Colomba P, Sciarrino S, Zizzo C, Francofonte D, Zora M, Scalia S, Brando C, Curto AL, Marsana EM, Olivieri R, Vitale S, Duro G (2020) Pompe disease: pathogenesis, molecular genetics and diagnosis. Aging (Albany NY) 12(15):15856–15874. 10.18632/aging.103794 32745073 10.18632/aging.103794 PMC7467391

[75] Duan D, Goemans N, Takeda S, Mercuri E, Aartsma-Rus A (2021) Duchenne muscular dystrophy. Nat Rev Dis Primers 7(1):13. 10.1038/s41572-021-00248-3 33602943 10.1038/s41572-021-00248-3 PMC10557455

[76] Xiong Q, Liu Y, Mo J, Chen Y, Zhang L, Xia Z, Yi C, Jiang S, Xiao N (2023) Gait asymmetry in children with Duchenne muscular dystrophy: evaluated through kinematic synergies and muscle synergies of lower limbs. Biomed Eng Online 22(1):75. 10.1186/s12938-023-01134-7 37525241 10.1186/s12938-023-01134-7 PMC10388506

[77] https://clinicaltrials.gov/study/NCT06124196?cond=muscular%20dystrophy&term=wearable%20device&rank=2

[78] Falzarano MS, Scotton C, Passarelli C, Ferlini A (2015) Duchenne Muscular Dystrophy: From Diagnosis to Therapy. Molecules 20(10):18168–18184. 10.3390/molecules201018168 26457695 10.3390/molecules201018168 PMC6332113

[79] Cesareo A, Nido SA, Biffi E, Gandossini S, D’Angelo MG, Aliverti A (2020) A Wearable device for breathing frequency monitoring: a pilot study on patients with muscular dystrophy. Sensors (Basel) 20(18):5346. 10.3390/s20185346 32961986 10.3390/s20185346 PMC7571149

[80] Maleki G, Zhuparris A, Koopmans I, Doll RJ, Voet N, Cohen A, van Brummelen E, Groeneveld GJ, De Maeyer J (2022) Objective Monitoring of Facioscapulohumeral Dystrophy During Clinical Trials Using a Smartphone App and Wearables: Observational Study. JMIR Form Res 6(9):e31775. 10.2196/31775.PMID:36098990;PMCID:PMC9516375 36098990 10.2196/31775 PMC9516375

[81] Mellion ML, Widholm P, Karlsson M, Ahlgren A, Tawil R, Wagner KR, Statland JM, Wang L, Shieh PB, van Engelen BGM, Kools J, Ronco L, Odueyungbo A, Jiang J, Han JJ, Hatch M, Towles J, Leinhard OD, Cadavid D (2022) Quantitative muscle analysis in FSHD using whole-body fat-referenced mri: composite scores for longitudinal and cross-sectional analysis. Neurology 99(9):e877–e889. 10.1212/WNL.0000000000200757 35750498 10.1212/WNL.0000000000200757

[82] Kurillo G, Chen A, Bajcsy R, Han JJ (2013) Evaluation of upper extremity reachable workspace using Kinect camera. Technol Health Care 21(6):641–656. 10.3233/THC-130764 24284552 10.3233/THC-130764

[83] Han JJ, de Bie E, Nicorici A, Abresch RT, Anthonisen C, Bajcsy R, Kurillo G, Mcdonald CM (2016) Reachable workspace and performance of upper limb (PUL) in duchenne muscular dystrophy. Muscle Nerve. 53(4):545–54. 10.1002/mus.24894 26342193 10.1002/mus.24894 PMC4779432

[84] de Bie E, Oskarsson B, Joyce NC, Nicorici A, Kurillo G, Han JJ (2017) Longitudinal evaluation of upper extremity reachable workspace in ALS by Kinect sensor. Amyotroph Lateral Scler Frontotemporal Degener 18(1–2):17–23. 10.1080/21678421.2016.1241278 27813421 10.1080/21678421.2016.1241278

[85] https://classic.clinicaltrials.gov/ct2/show/NCT04003974

[86] Gerhalter T, Müller C, Maron E, Thielen M, Schätzl T, Mähler A, Schütte T, Boschmann M, Herzer R, Spuler S, Gazzerro E (2023) “suMus,” a novel digital system for arm movement metrics and muscle energy expenditure. Front Physiol 26(14):1057592. 10.3389/fphys.2023.1057592 10.3389/fphys.2023.1057592 PMC9909604 36776973

[87] https://clinicaltrials.gov/study/NCT06089018?term=Observational%20Study%20of%20Digital%20Biomarkers%20of%20Myotonia%20and%20Gait%20in%20Adults%20and%20Children%20With%20Myotonic%20Dystrophy&rank=1

[88] Ricotti V, Kadirvelu B, Selby V, Festenstein R, Mercuri E, Voit T, Faisal AA (2023) Wearable full-body motion tracking of activities of daily living predicts disease trajectory in Duchenne muscular dystrophy. Nat Med. 29(1):95–103. 10.1038/s41591-022-02045-1 36658421 10.1038/s41591-022-02045-1 PMC9873561

[89] Milazzo M, Spezzaneve A, Astrea G, Giorgolo F, Tonacci A, Sansone F, Calderisi M, Ingene Group (2021) AUTOMA: a wearable device to assess the upper limb muscular activity in patients with neuromuscular disorders. Acta Myol 40(4):143–151. 10.36185/2532-1900-057 35047754 10.36185/2532-1900-057 PMC8744014

[90] Baeza-Barragán MR, Labajos Manzanares MT, Amaya-Álvarez MC, Morales Vega F, Rodriguez Ruiz J, Martín-Valero R (2023) Effectiveness of a 5-week virtual reality telerehabilitation program for children with duchenne and becker muscular dystrophy: prospective quasi-experimental study. JMIR Serious Games 15(11):e48022. 10.2196/48022 10.2196/48022 PMC10686615 37990809

[91] https://classic.clinicaltrials.gov/ct2/show/NCT06138639

[92] https://clinicaltrials.gov/study/NCT05876780?cond=Beta-Sarcoglycan%20Deficiency&rank=2

[93] Regulation (EU) 2016/679 of the European Parliament and of the Council of 27 April 2016 on the Protection of Natural Persons with Regard to the Processing of Personal Data and on the Free Movement of Such Data, and Repealing Directive 95/46/EC (General Data Protection Regulation) (Text with EEA Relevance). Available online: https://eur-lex.europa.eu/legal-content/EN/TXT/?uri=CELEX:32016R0679

